# An Inter-observer Study to Determine Radiotherapy Planning Target Volumes for Recurrent Gynaecological Cancer Comparing Magnetic Resonance Imaging Only With Computed Tomography-Magnetic Resonance Imaging

**DOI:** 10.1016/j.clon.2021.02.003

**Published:** 2021-05

**Authors:** D. Bernstein, A. Taylor, S. Nill, G. Imseeh, G. Kothari, M. Llewelyn, K.N. De Paepe, A. Rockall, A.-M. Shiarli, U. Oelfke

**Affiliations:** ∗ Joint Department of Physics, The Institute of Cancer Research and The Royal Marsden NHS Foundation Trust, London, UK; † Gynaecology Unit, Royal Marsden NHS Foundation Trust, London, UK; ‡ Joint Department of Physics, The Institute of Cancer Research and The Royal Marsden NHS Foundation Trust, Sutton, London, UK; § Radiotherapy and Imaging, The Institute of Cancer Research and The Royal Marsden NHS Foundation Trust, Sutton, London, UK; ¶ Peter MacCallum Cancer Center, Melbourne, Victoria, Australia; || Department of Radiology, Cambridge University Hospitals NHS Foundation Trust, Cambridge, UK; ∗∗ Department of Radiology, Royal Marsden NHS Foundation Trust, London, UK; †† Department of Surgery and Cancer, Imperial College London, London, UK

**Keywords:** Delineation uncertainty, planning target volume, radiotherapy, recurrent gynaecological cancer

## Abstract

**Aims:**

Target delineation uncertainty is arguably the largest source of geometric uncertainty in radiotherapy. Several factors can affect it, including the imaging modality used for delineation. It is accounted for by applying safety margins to the target to produce a planning target volume (PTV), to which treatments are designed. To determine the margin, the delineation uncertainty is measured as the delineation error, and then a margin recipe used. However, there is no published evidence of such analysis for recurrent gynaecological cancers (RGC). The aims of this study were first to quantify the delineation uncertainty for RGC gross tumour volumes (GTVs) and to calculate the associated PTV margins and then to quantify the difference in GTV, delineation uncertainty and PTV margin, between a computed tomography-magnetic resonance imaging (CT-MRI) and MRI workflow.

**Materials and methods:**

Seven clinicians delineated the GTV for 20 RGC tumours on co-registered CT and MRI datasets (CT-MRI) and on MRI alone. The delineation error, the standard deviation of distances from each clinician's outline to a reference, was measured and the required PTV margin determined. Differences between using CT-MRI and MRI alone were assessed.

**Results:**

The overall delineation error and the resulting margin were 3.1 mm and 8.5 mm, respectively, for CT-MRI, reducing to 2.5 mm and 7.1 mm, respectively, for MRI alone. Delineation errors and therefore the theoretical margins, varied widely between patients. MRI tumour volumes were on average 15% smaller than CT-MRI tumour volumes.

**Discussion:**

This study is the first to quantify delineation error for RGC tumours and to calculate the corresponding PTV margin. The determined margins were larger than those reported in the literature for similar patients, bringing into question both current margins and margin calculation methods. The wide variation in delineation error between these patients suggests that applying a single population-based margin may result in PTVs that are suboptimal for many. Finally, the reduced tumour volumes and safety margins suggest that patients with RGC may benefit from an MRI-only treatment workflow.

## Highlights

• The first paper to focus on recurrent gynaecological tumour radiotherapy targets
• Presents values for delineation error and the corresponding safety margins
• Standard methods produce margins larger than those reportedly used elsewhere
• Using MRI alone results in smaller target volumes than using CT and MRI together

## Introduction

Radiotherapy, in combination with chemotherapy, has been recommended for the treatment of locoregional recurrence cervical and endometrial cancers in patients previously treated with radical surgery [[1], [2], [3], [4]]. This usually involves a combination of external beam radiotherapy to the pelvis followed by a boost to deliver a high dose to macroscopic disease with either brachytherapy or external beam radiotherapy if brachytherapy is not feasible. The tumour dose is often limited by the risk of toxicity due to close proximity to bowel, bladder and rectum.

Despite significant advances in radiotherapy techniques, geometric uncertainties persist in radiotherapy, with the largest arguably being the uncertainty in tumour delineation [[5], [6], [7]], here referred to as delineation uncertainty.

Radiotherapy treatments are based on a set of treatment volumes. First macroscopic disease is delineated on imaging as the gross demonstrable tumour mass, known as the gross tumour volume (GTV). From this, the clinical target volume (CTV) is derived, to account for potential microscopic spread, although when delivering external beam radiotherapy or brachytherapy boost for recurrent gynaecological cancers (RGC), the CTV may be the same as the GTV. Residual geometric uncertainties are then accounted for by adding safety margins to the CTV, resulting in the planning target volume (PTV), to which the treatment is planned.

A widely used method for calculating PTV margins is to use the van Herk margin recipe [8]. This requires each source of geometric uncertainty to be quantified, such as those associated with tumour delineation. According to that approach, failure to quantify these uncertainties can result in suboptimal PTVs and consequently treatments, where margins that are too small correspond with a lower than intended chance of the tumour receiving the planned dose, whereas margins that are too big result in unnecessary radiation dose to surrounding healthy tissue, which can lead to additional toxicities. For pelvic recurrence of gynaecological cancer, tumours can vary significantly between patients in volume, location and relation to the organs at risk. To the authors' knowledge, although required for PTV margin calculation, there is no published literature that quantifies the delineation uncertainty for RGC in a way that can be used for margin calculations. The use of different imaging modalities is known to affect the magnitude of delineation uncertainty [7,[9], [10], [11], [12]]. Due to the superior soft-tissue differentiation with magnetic resonance imaging (MRI) over computed tomography (CT), current practice is to delineate RGC GTVs using co-registered CT and MRI images, henceforth referred to as CT-MRI. Recent technological advances, such as MRI-only based treatment planning [13], are leading to the possibility of treatments based on MRI images alone.

The aims of this study were first to quantify delineation uncertainty for RGC GTVs and to calculate the associated PTV margins and then to quantify the difference in GTV, delineation uncertainty and PTV margin, between a CT-MRI and MRI workflow.

## Materials and Methods

### Delineation Study

A retrospective inter-observer variability study was carried out to quantify the GTV delineation uncertainty from patients with RGC in the central pelvis. This study was approved by the appropriate Institutional Review Board (SE433) and written informed consent was obtained from all subjects.

Twenty patients with RGC in the central pelvis, having previously had a hysterectomy, were retrospectively selected for this study on the basis of having a treatment GTV >10 cm^3^, and with both CT and MR images available.

CT scans were carried out on a 16-slice scanner, with a 2.5 mm slice separation for 17 of the patients, 1 mm for two patients and 1.3 mm for one patient. MRI scans were carried out on 1.5 Tesla scanners acquired at a range of institutions. All patients had a T2-weighted turbo spin echo sequence acquired axially, except for patient 13 who had a T2-weighted three-dimensional space sequence. MRI slice separations depended on the clinical need at the time of scanning, ranging from 3.0 to 6.0 mm (mean ± standard deviation = 4.5 ± 1.1 mm).

Seven clinicians from a single institution participated in the study: two radiologists and two radiation oncologists specialising in pelvic radiotherapy with at least 4 years' experience, and three radiation oncologists training in pelvic radiotherapy with 1–3 years' experience. Each clinician delineated the GTV on each of the patient scans using local clinical protocols.

To avoid the CT information biasing the MRI-only outlines, delineations were first performed on MRI alone, followed by CT-MRI registered using rigid registration applied to the local soft-tissue anatomy around the GTV. Registrations were carried out by an experienced medical physicist, and then reviewed by a consultant radiation oncologist. Each of the observers was blinded to all other delineations by having their own dedicated image dataset and password to the contouring software. Delineations were carried out on the Eclipse Treatment Planning System, version 13.6 (Varian Medical Systems, Palo Alto, CA, USA).

### Delineation Error (Σ_D_)

To calculate the GTV–PTV margin required at each point, delineation uncertainty must be quantified as the delineation error (Σ_D_). This is the standard deviation of the distances from a reference outline to each outline. It is calculated using equation 1, where *d*_*i*_ is the distance from the reference outline to the *i*th observer's outline, *N*_o_ is the number of observers and $\bar{d}$ is the mean distance. Although many metrics for measuring observer variability are used [7,14,15], Σ_D_ is the only one that can be used in a traditional PTV margin recipe to calculate PTV margins.

(1) $$\Sigma_{D}\left(d\right)=\sqrt{\frac{1}{N_{O}-1}\overset{N_{O}}{\underset{i=1}{\sum }}{\left(d_{i}-\bar{d}\right)}^{2}}$$

To measure Σ_D_ for each point in a patient, the approach taken by Deurloo *et al.* [16] was followed. First, the two-dimensional contour sets were converted to three-dimensional surfaces comprising of vertices and faces. A reference surface for each patient was then generated from all the clinician's GTV surfaces. This was carried out using the simultaneous truth and performance level estimation (STAPLE) algorithm [17] at a 95% confidence level. This produced a surface that encompasses voxels that have a 95% or more probability of belonging to the GTV based on the provided outlines. These consensus surfaces were generated using Computational Environment for Radiological Research (CERR) software module [18] implemented within MatLab (The MathWorks Inc., Natick, MA, USA).

For the *j*th vertex on the reference surface, the reference surface normal vector, **n**_**j**_, was determined. The distance along that normal vector to each comparator surface was then measured and equation 1 used to give the delineation error at the *j*th vertex Σ_D,j_. The result of this process is a three-dimensional surface map with potentially varying values of Σ_D_.

**Fig 1:**
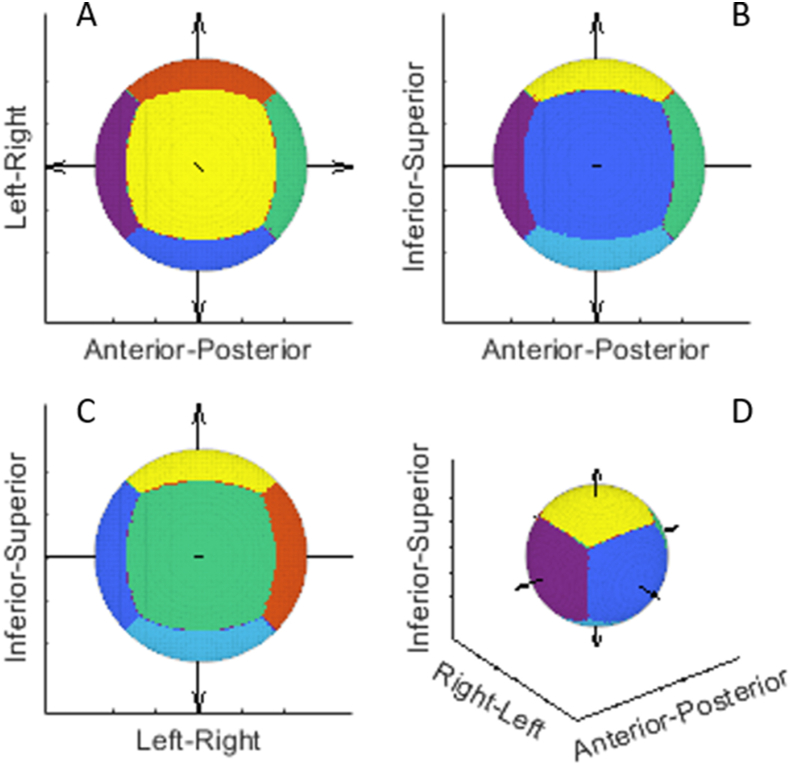
Illustrations of the vertex classifications for a spherical target as either left (blue), right (orange), anterior (purple), posterior (green), superior (yellow) or inferior (light blue). Arrows represent the axes from the centre of the volume. (A) is viewed from the superior axis, (B) from the left axis, (C) from the posterior axis and (D) is an oblique view.

PTV margin recipes require summary Σ_D_ values for each sector of the GTV corresponding to each of the cardinal axes. To determine which sector each vertex on the consensus surface belongs to, vector *K*_j_, which originated at the centre of the consensus surface and terminated at vertex v_j_, was created. As were six vectors originating at the centre of the volume and pointing parallel to the patient left ($\lambda_{\text{L}}$), right ($\lambda_{\text{R}}$), anterior ($\lambda_{\text{A}}$), posterior ($\lambda_{\text{P}}$), superior ($\lambda_{\text{S}}$) and inferior ($\lambda_{\text{I}}$) axes. The angles between *K*_j_ and each of the λ vectors were then measured. Finally, the vertex v_j_ was assigned to a single sector corresponding to the vector λ with the smallest angle to *K*_j_. This process was repeated for each vertex on the consensus surface. Figure 1 illustrates the results for a spherical target. The approach of classifying the vertices in this way is similar to one taken in previous studies [[19], [20], [21]], however those publications only measured points along the cardinal planes, unlike this study where classifications and measurements were carried out for the whole surface.

The overall Σ_D_ for a single patient was given by the geometric mean of Σ_D_, calculated over all vertices in that patient. The overall Σ_D_ for all patients combined was then given by the geometric mean of Σ_D_ from each patient. For values given for a single sector, these measurements and calculations were carried out for each sector separately. Pooling standard deviations in this way assumes that the variances between each group are equal; this assumption was tested using the methods described below. It was assumed that each case had an equal number of samples to avoid the largest GTVs dominating the value of Σ_D_s for all patients combined.

### Gross Tumour Volume–Planning Target Volume Margin Calculation

The GTV–PTV margin, *m*, was calculated for each scenario using the van Herk margin recipe [8] shown in equation 2. This is based on ensuring the CTV, which is identical to the GTV in this case, is covered by the 80% isodose, as can be the case in stereotactic body radiotherapy, for 90% of the patients. Σ and σ are the combined standard deviation of all the systematic and random errors, respectively. Here it was assumed that daily image guidance was used and that random errors totalled 1 mm, as did systematic errors (excluding target delineation), based on a study by McNair *et al.* [22]. With this magnitude of random errors, the margins required for coverage by the 95% isodose would be 0.3 mm larger.

(2) m=2.5∑+0.4σ

### Statistical Analysis

The Wilcoxon signed rank test was used to compare differences in tumour volumes between CT-MRI and MRI. The Brown-Forsythe test was used to assess differences in the variance of the distances, Σ_D_^2^, between patients, imaging conditions and to determine the validity of pooling standard deviation data.

All tests were two-tailed and with significance level of 0.05. *P*-values were modified to compensate for multiple comparisons using the Bonferroni-Holm correction. All analyses and data processing were carried out in MatLab R2019a.

## Results

The primary diagnosis for the 20 cases was: eight (40%) patients with carcinoma of the endometrium, five (25%) with cervical, four (20%) with vaginal and three (15%) with ovarian carcinoma. All patients had previously undergone surgery including total hysterectomy.

### Gross Tumour Volumes

**Fig 2:**
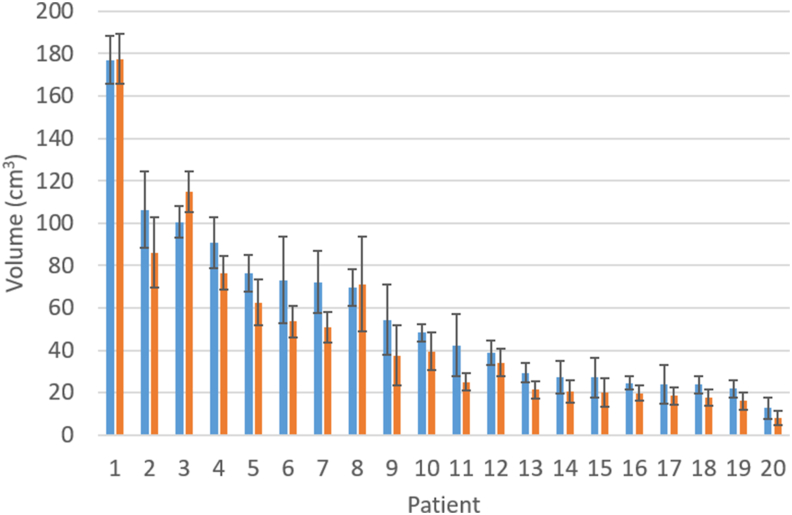
The mean gross tumour volume (GTV; bar height) and the standard deviation (error bars) for each patient, measured over all observers. Computed tomography–magnetic resonance imaging (CT-MRI) volumes are shown in blue, MRI only in orange. Patients are ordered by descending CT-MRI volume.

The mean and standard deviation of the GTV for each patient, taken over all observers, are illustrated in Figure 2, which shows a large range in volumes. The overall mean ± standard deviation was 57.0 ± 40.5 cm^3^ for CT-MRI and 48.5 ± 41.5 cm^3^ for MRI alone. MRI volumes were smaller than CT-MRI (*P* < 0.001), by an average of 15%.

### Delineation Error (Σ_D_) and PTV Margins (m)

**Fig 3:**
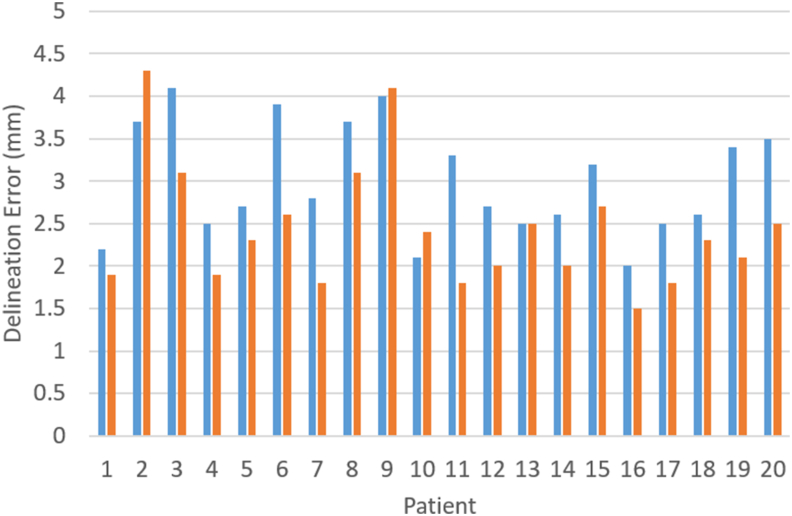
Delineation error (Σ_D_) for each patient for both computed tomography–magnetic resonance imaging (CT-MRI; blue) and MRI alone (orange).

The measured Σ_D_ are presented in Table 1 for all patients combined. Overall, Σ_D_ was smaller when using MRI alone relative to CT-MRI for both the whole surface and for each sector (*P* < 0.001). Σ_D_ was significantly different within and between patients and sectors for both imaging conditions (*P* < 0.001). Σ_D_ for each patient is illustrated in Figure 3, which also shows MRI Σ_D_ to be smaller in 16 of the 20 cases compared with CT-MRI. The PTV margins resulting from the overall measured Σ_D_ are also presented in Table 1. The overall Σ_D_ and the resultant margins grouped by primary diagnosis are presented in Table 2. These are smaller for MRI relative to CT-MRI due to the smaller MRI measured Σ_D_ irrespective of tumour volume.

**Table 1:** Delineation error (Σ_D_) pooled over all patients and observers, measured over the whole surface and for each anterior (A), posterior (P), left (L), right (R), superior (S) and inferior (I) sectors. Resultant gross tumour volume–planning target volume (GTV–PTV) margins, m, are also presented. Values are shown for both computed tomography–magnetic resonance imaging (CT-MRI) and MRI alone

	Whole surface	Sector					
		A	P	L	R	S	I
Σ_D_ (mm) CT-MRI	3.1	2.7	2.6	2.8	3.0	3.2	3.6
Σ_D_ (mm) MRI	2.5	2.0	2.4	2.2	2.6	3.0	2.7
m (mm) CT-MRI	8.5	7.6	7.4	7.8	8.3	8.8	9.7
m (mm) MRI	7.1	6.0	6.9	6.4	7.4	8.3	7.6

**Table 2:** Delineation error (Σ_D_) and resultant gross tumour volume–planning target volume (GTV–PTV) margins, m, for patients grouped by primary diagnosis. Values are shown for both computed tomography–magnetic resonance imaging (CT-MRI) and MRI alone

Primary diagnosis	Σ_D_ (mm)		m (mm)	
	CT-MRI	MRI	CT-MRI	MRI
Ovary	2.4	2.2	7.0	6.5
Endometrium	3.1	2.6	8.5	7.2
Cervix	2.7	2.0	7.6	6.0
Vagina	3.6	2.9	9.7	8.0

**Fig 4:**
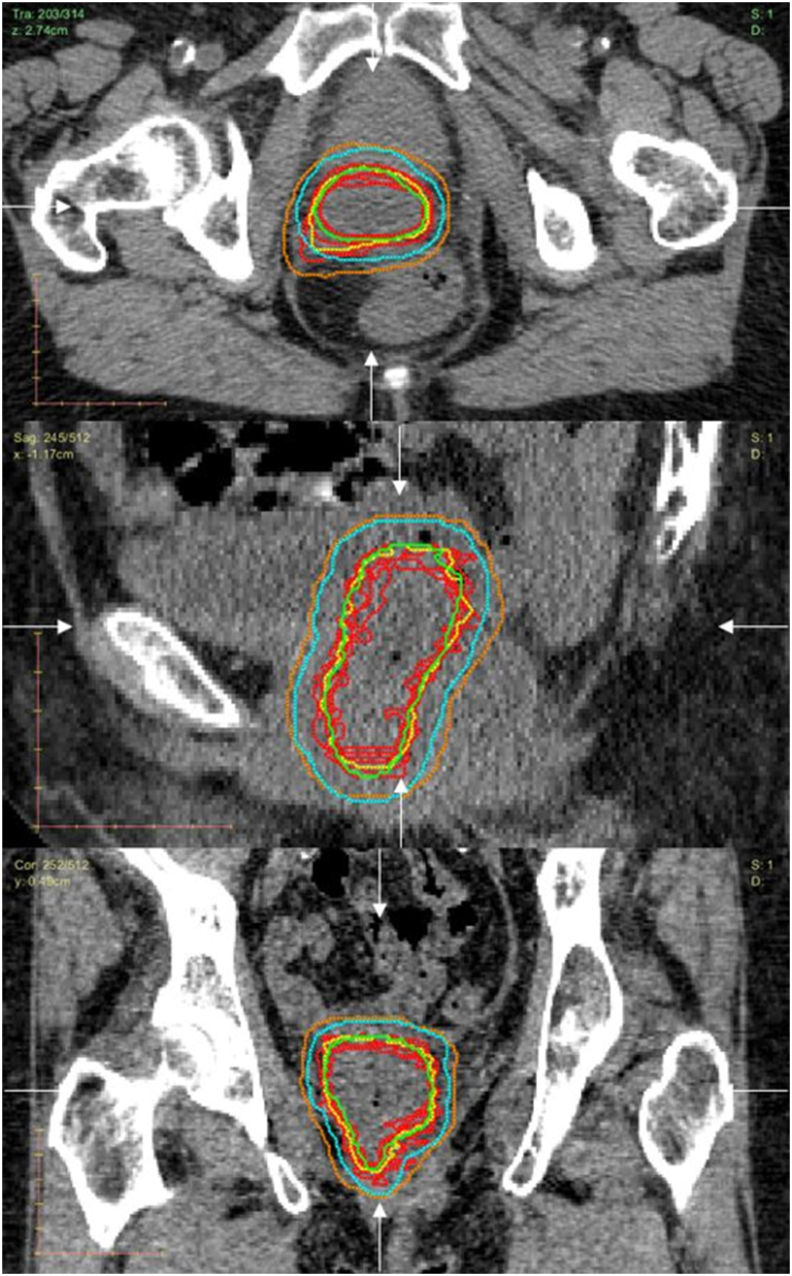
A selection of the outlines for patient 14; computed tomography–magnetic resonance imaging (CT-MRI) gross tumour volume (GTV) outlines from each observer are shown in red; CT-MRI GTV consensus outline is shown in yellow; MRI consensus outline is shown in green; CT-MRI planning target volume (PTV) is shown in orange; finally the MRI PTV is shown in turquoise. The locations of the viewing planes are indicated by the white arrows.

To illustrate the typical differences in outlines between observers, Figure 4 shows the CT-MRI outlines for patient 14, who had Σ_D_ equal to the group median of 2.7 mm. The CT-MRI and MRI consensus STAPLE outlines are also shown, as are the PTVs grown from them.

## Discussion

This is the first paper to present an inter-observer variability study for RGC GTVs and to quantify the variability using the delineation error metric. These data are important as they are needed to calculate PTV margins using standard methods such as the van Herk margin recipe [8]. Our results provide a reference set of values against which other institutions can compare. Despite this benefit of measuring delineation error, few studies do so when performing inter-observer variability studies [7,15], making comparisons difficult. To give context to these results, the overall measured 3.1 mm delineation error is larger than the 2.0 mm for the prostate as measured by Alasti *et al.* [23] using CT alone, but is smaller than the average 5.5 mm value measured for the prostate bed by Ost *et al.* [24].

In addition to the delineation error varying between the sites of primary diagnosis, it also varied widely between patients, with Σ_D_ ranging from 2.0 to 4.1 mm for CT-MRI and 1.5–4.3 mm for MRI. These values were found to be statistically different between patients, suggesting that the delineation error for this cohort is not sufficiently homogeneous to be accurately combined into an overall value. These results bring into question whether population-based GTV–PTV margins are appropriate for RGC GTVs. Using population-based margins for these patients will likely result in target volumes that are either too large or too small for many patients. These patients will probably benefit from a more personalised approach to account for delineation uncertainty, for example through performing an inter-observer variability study for each patient. However, this is unlikely to be feasible in a clinical setting. An alternative solution, developed by Bernstein *et al*. [33], derives personalised PTVs from outlines produced by a single clinician. One of the few studies that assess this variation between patients is Steenbakkers *et al.* [25], who reported the delineation error to range from 1.9 to 7.5 mm for lung GTVs based on CT-PET images. In our study, the variation in MRI resolution between patients, primarily in the axial slice separation, is probably responsible for some of the variation seen between patients. Using consistent MRI parameters with high resolution is likely to improve consistency, delineation error and reduce superior–inferior PTV margins. However, it is unlikely to affect the findings in this study, particularly when considering that the delineation error was of a similar magnitude axially and varied significantly between patients.

The overall CT-MRI margin of 8.5 mm is larger than the margins reported in the literature for RGC, or similar, treatment sites. For example, Dewas *et al.* [26] reported using 3 mm GTV–PTV margins for lateral pelvic recurrences. A publication by Park *et al.* [27], which reported on the experience of three centres in Korea treating recurrent or oligometastatic uterine cervix cancer patients, reported all centres also using 3 mm margins, as did Kunos *et al.* [28], who reported the use of 3 mm margins in a phase II trial of metastatic gynaecological malignancies. A publication by Hasan *et al.* [29] into the treatment of recurrent gynaecological malignancies reported using 3 mm margins in most patients, albeit with a range of 0–5 mm. These publications did not state how their margins were determined. However, to achieve a margin of 3 mm using the van Herk margin recipe would require a Σ_D_ of approximately 1 mm. Yet, the smallest value seen for Σ_D_ in this study evaluating 20 patients was 2 mm for CT-MRI.

Although this study focused on external beam radiotherapy, the delineation uncertainty and variability shown is probably also present for brachytherapy treatments. In brachytherapy, GTV–PTV margins are not routinely used, therefore these uncertainties may have a detrimental impact on brachytherapy treatments.

The use and consequences of inadequate margins in the pelvis has been reported by Engels *et al.* [30] for patients with prostate cancer. The authors reported significantly worse 5-year freedom from biochemical failure in patients treated with 3–5 mm PTV margins than those treated with 6–10 mm margins, highlighting the importance of optimal target coverage. The effect of inadequate PTV margins on clinical outcomes in patients with RGC warrants further investigation.

To assess the potential benefit of recent technological developments, such as synthetic CT, as described by Johnstone *et al.* [31], the impact of RGC GTV delineation on MRI alone was compared with CT-MRI. MRI-based tumour volumes were smaller for most patients, by an average of 15%. This is similar to the 10% and 18% found by Alasti *et al.* [23] and Gunnlaugsson *et al.* [32] respectively for the prostate when using MRI alone. RGC GTV delineation error was also smaller, by 0.6 mm overall, which translated into a smaller PTV margin. In comparison, Alasti *et al.* [23] measured a 0.2 mm delineation error reduction for the prostate. One possible explanation for this is anatomical changes between the two scans, for example due to target and/or organ at risk movement, leading to differences in GTV boundary position between the two images, imperfect registration and ultimately increasing uncertainty when using multiple scans. Assuming no significant geometric uncertainties are introduced by an MRI-only workflow, the combination of smaller volumes and lower delineation error would result in smaller PTVs.

In conclusion, this study is the first to quantify delineation error for RGC GTVs. Presenting the inter-observer variability in terms of delineation error has allowed the PTV margins to be calculated, which are larger than those reported in the literature for similar disease sites. A wide range in delineation error was found between patients, which corresponded to a wide range in the theoretical GTV-PTV margins, suggesting that one margin for all cases is unlikely to be a viable strategy. Finally, the smaller delineation error and GTVs suggest that patients with RGC may benefit from an MRI-only treatment workflow. This study highlights the need for further research into the PTV margins and imaging modalities used for patients with RGC treated with radiotherapy.

## Conflicts of interest

The authors declare no conflicts of interest.
